# Recent trends in dual-acting hybrid antibiotics and combination therapies against Gram-negative pathogens

**DOI:** 10.71150/jm.2601004

**Published:** 2026-03-31

**Authors:** Ji Eun Son, Umji Choi, Gyubin Han, Jeongho Lee, Chang-Ro Lee

**Affiliations:** Department of Biological Sciences, Myongji University, Yongin 17058, Republic of Korea

**Keywords:** hybrid antibiotic, combination therapy, synergy, Gram-negative pathogen, molecular mechanism

## Abstract

Antibiotic resistance poses a serious challenge to public health worldwide; however, the development of new antibiotic classes for combating bacterial infections, especially those caused by Gram-negative pathogens, has slowed in recent years. Dual-acting hybrid antibiotics with a metabolically non-cleavable covalent bond represent an emerging strategy for developing novel antibiotic classes to overcome antibiotic resistance. The covalent connection between two antibiotics results in a fixed pharmacokinetic profile of a single molecule and can impede bacterial efflux. However, as most antibiotics do not have membrane-destabilizing activity, the resulting increase in molecular weight by connection of two antibiotics could limit their activity against Gram-negative bacteria, whose outer membrane forms a strong barrier blocking the penetration of high-molecular weight antibiotics. Here, we review recent developments in dual-acting hybrid antibiotics targeting Gram-negative bacteria, with a focus on their antibacterial efficacy. We also discuss combination therapy strategies in which the underlying molecular mechanisms of synergy have been characterized. Finally, we outline future directions for the rational design of hybrid antibiotics against Gram-negative pathogens.

## Introduction

Antibiotics have been central to clinical medicine since the second half of the 20th century; however, the evolution and spread of antibiotic resistance in pathogenic bacteria pose a serious threat to global public health (Lee et al., 2013). The World Health Organization (WHO) has identified six pathogens as high-priority pathogens because of their serious multidrug resistance abilities. They are collectively known as ESKAPE pathogens (*Enterococcus faecium*, *Staphylococcus aureus*, *Klebsiella pneumoniae*, *Acinetobacter baumannii*, *Pseudomonas aeruginosa*, and *Enterobacter* spp.). In 2019, bacterial antimicrobial resistance was directly linked to 1.27 million deaths, and approximately 95% of these deaths were associated with the six pathogens, including *A. baumannii*, *E. coli*, *K. pneumoniae*, *P. aeruginosa*, and *S. aureus* (Antimicrobial Resistance Collaborators, 2022). Among these, all except *S. aureus* are Gram-negative bacteria.

Bacteria can easily decrease their susceptibility to antibiotics through several mechanisms, including horizontal gene transfer and mutations (Thomas and Nielsen, 2005). Consequently, new weapons for combating bacterial infections are continually required. Despite this, only six new antibiotic classes have been clinically approved in the past 20 years, mostly discovered between the 1930s and the 1960s (Butler et al., 2017). Importantly, none of these classes are particularly effective in the treatment of infections caused by Gram-negative pathogens, such as *A. baumannii*, *E. coli*, *K. pneumoniae*, and *P. aeruginosa* (Butler et al., 2017). To overcome this decline in antibiotic discovery, various alternate strategies are currently being explored.

**Fig. 1.**
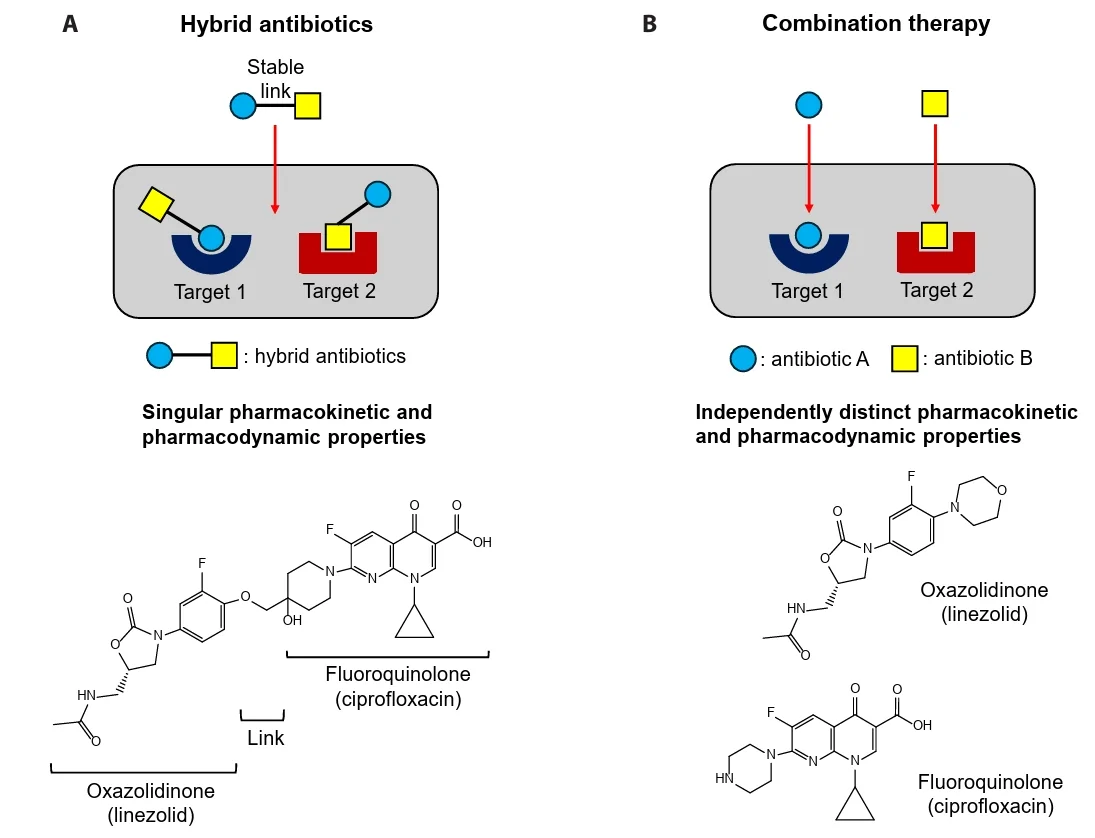
Comparison of hybrid antibiotics with combination therapy. Both strategies can broaden the spectrum of action, reduce antibiotic resistance, and induce synergistic effects. (A) Hybrid antibiotics containing a metabolically non-cleavable covalent linker demonstrate singular pharmacokinetic and pharmacodynamic properties. A representative example of hybrid antibiotics is shown. (B) Combination therapy demonstrates independent, and potentially unpredictable, pharmacokinetic and pharmacodynamic properties. A representative example of combination therapy is shown.

The development of dual-acting hybrid antibiotics has emerged as a promising strategy. These hybrids are constructed by ligating two antibiotics together or antibiotics and non-antibiotic moieties such as siderophores, adjuvants, or membrane-active peptides, via a metabolically stable linker, thereby forming a single heterodimer. In the following, we focus exclusively on hybrid antibiotics constructed by ligating two antibiotics together. In principle, such a heterodimeric entity should retain the antibacterial activity of its two constituent pharmacophores. In contrast with combination therapy, in which the two drugs retain independently distinct pharmacokinetic and pharmacodynamic properties, hybrid antibiotics connected by a covalent linker demonstrate fixed pharmacokinetic and pharmacodynamic profiles as a single molecular entity (Fig. 1). Additionally, covalent linkage between two antibiotics can impede bacterial efflux and reduce the development of resistance and toxicity when administrated in vivo (Theuretzbacher, 2020).

Despite the various advantages of hybrid antibiotics with metabolically non-cleavable bonds, they have serious disadvantages when used to treat infections caused by Gram-negative pathogens. These pathogens have an additional outer membrane that blocks the penetration of antibiotics with high molecular weights (O'Shea and Moser, 2008). As most antibiotics do not have outer membrane-destabilizing activity exhibited by colistin, an increase in molecular weight due to the covalent linkage between two antibiotics is a serious limitation of hybrid antibiotics. Indeed, most hybrid antibiotics with dual mechanisms mainly target Gram-positive bacteria (Koh et al., 2023; Pokrovskaya and Baasov, 2010; Theuretzbacher, 2020). While it is true that molecular weight influences uptake of hybrid antibiotics, other physicochemical factors, including polarity, charge distribution, conformational flexibility, amphiphilicity, and susceptibility to efflux, are also important. Therefore, modification of physicochemical properties can enhance the penetration of hybrid antibiotics.

In this review, we summarize recent advances in dual-acting hybrid antibiotics designed to target Gram-negative bacteria and discuss their antibacterial activities. Additionally, we explore recent examples of combination therapy in which the molecular mechanisms underlying the synergistic effects have been elucidated. Finally, we discuss future research directions for the development of hybrid antibiotics targeting Gram-negative bacteria.

## Quinolone-Containing Antibiotic Hybrids

Quinolone-containing antibiotic hybrids are the most comprehensively represented hybrid antibiotics. The structure–activity relationships of quinolones has been widely studied and is well defined. The amine group in the C-7 piperidino moiety is a readily acceptable and tolerated position; therefore, it can be used for the attachment of bulky compounds via N-alkylation, carbamate formation, or quaternary salt formation. Additionally, quinolones are chemically stable under various synthetic conditions, facilitating their conjugation with other antibiotics. Ouinolones target two enzymes, DNA gyrase and topoisomerase IV, which is another advantage of this antibiotic. Steric hinderance imposed by the attachment of a partner antibiotic or linker can inhibit interaction with the target protein. In the case of quinolones, this limitation can be compensated by interaction with a second target protein. Nevertheless, quinolones have two disadvantages: rapid resistance development due to spontaneous mutations of target enzymes, and limited availability of antibiotics showing the synergy effect with quinolones in combination therapy.

Among quinolone antibiotics, ciprofloxacin has been extensively used to construct dual-acting antibiotic hybrids (Table 1). Cephalosporin–ciprofloxacin hybrids have been developed (Evans et al., 2019), functioning as dual-acting antibiotics in the absence of β-lactamase, and as prodrugs in its presence. β-Lactamase-mediated hydrolysis of cephalosporins results in the intracellular release of ciprofloxacin, restoring antimicrobial efficacy (Evans et al., 2019). Accordingly, these compounds have exhibited good antimicrobial activity against New Delhi metallo-β-lactamase-expressing *E. coli* (Table 1).

The ciprofloxacin pharmacophore has also been used to synthesize ciprofloxacin–aminoglycoside hybrid compounds (Pokrovskaya et al., 2009; Shavit et al., 2017). A series of hybrids linking ciprofloxacin and neomycin via a 1,2,3-triazole moiety have been designed and synthesized (Pokrovskaya et al., 2009). Several of these hybrids are significantly more potent than the parent neomycin and showed good antimicrobial activity against neomycin-resistant *E. coli* (Table 1). More recently, kanamycin–ciprofloxacin hybrid compounds have been reported (Shavit et al., 2017). Their minimal inhibitory concentrations (MICs) against *E. coli* are comparable to those of neomycin–ciprofloxacin hybrids (Table 1). Notably, these dual-target hybrids have significantly lower resistance frequencies in *E. coli* than each individual drug or their combination (Shavit et al., 2017). Among the aminoglycosides, tobramycin shows concentration-dependent killing against *P. aeruginosa* at high concentrations by increasing outer membrane permeability through lipopolysaccharide destabilization (Bulitta et al., 2015; Dhiman et al., 2023; Herzog et al., 2012). Consequently, tobramycin–ciprofloxacin hybrids show strong synergy with several fluoroquinolones against multidrug-resistant *P. aeruginosa* by enhancing fluoroquinolone penetration (Dhiman et al., 2023; Gorityala et al., 2016). These findings indicate that tobramycin-based hybrids may represent a novel class of antibiotic potentiators.

A series of hybrid antibiotics containing ciprofloxacin and macrolides, such as erythromycin and telithromycin, have also been synthesized (Hutinec et al., 2010; Ma et al., 2019). These compounds have exhibited good antibacterial activity against *Haemophilus influenzae*, *Mycoplasma pneumoniae*, *Moraxella catarrhalis*, and *E. coli* (Table 1). A recent study reported a hybrid compound with a relatively low MIC (8 μg/ml) against erythromycin-resistant *M. pneumoniae* (Liu et al., 2022) (Table 1).

Fluoroquinolone–oxazolidinone hybrids are among the most extensively studied fluoroquinolone-based dual-acting antibiotic hybrids (Table 1), and several of these compounds have been reported (Hubschwerlen et al., 2003a, 2003b; Liu et al., 2019; Scaiola et al., 2019). Oxazolidinone antibiotics, such as linezolid and tedizolid, block the initiation of protein synthesis by binding to the peptidyl site of the ribosomal 50S subunit (Bozdogan and Appelbaum, 2004). However, owing to the activity of efflux pumps, most Gram-negative bacteria show intrinsic resistance to oxazolidinones (Schumacher et al., 2007). Interestingly, most of the synthesized fluoroquinolone–oxazolidinone hybrids generally exhibit good antibacterial activity against *E. coli* and *H. influenzae* (Table 1), but show only moderate activity against *P. aeruginosa* (Liu et al., 2019).

Another class of novel fluoroquinolone-based hybrids involves conjugation to the macrocyclic core of ansamycin antibiotics, such as rifampicin and kanglemycin (Peek et al., 2022; Yuan et al., 2020). These antibiotics block mRNA synthesis in bacteria by inhibiting bacterial DNA-dependent RNA polymerase (Calvori et al., 1965). Several of the resulting hybrids have demonstrated good antibacterial activity against *E. coli*, *H. influenzae*, and *Helicobacter pylori* (Table 1). Notably, one hybrid compound, TNP-2092, displayed a significantly low MIC (0.06 μg/ml) against *Neisseria gonorrhoeae* (Yuan et al., 2020).

Recently, ciprofloxacin-based hybrids fused to sulfonamides, which inhibit bacterial dihydropteroate synthetase in the folic acid pathway (Kratky et al., 2012), have been described (Ibrahim et al., 2022). These compounds exhibit good antibacterial activity against *E. coli* (Ibrahim et al., 2022). Another antifolate antibiotic, trimethoprim, which binds to dihydrofolate reductase and inhibits the reduction of dihydrofolic acid to tetrahydrofolic acid (Brogden et al., 1982), has been fused with ciprofloxacin (Karoli et al., 2012). One resulting hybrid compound has shown good antibacterial activity against *P. aeruginosa* and *K. pneumoniae*, but lacks activity against trimethoprim- and ciprofloxacin-resistant *E. coli* (Karoli et al., 2012).

Metronidazole is active against most anaerobic and microaerophilic bacteria, but lacks activity against most aerobic bacteria, including *E. coli* (Leitsch, 2019). Several quinolone-based hybrids incorporating metronidazole have been synthesized (Cui et al., 2014; Zhang et al., 2015), and most have shown good antibacterial activity against *P. aeruginosa* and *E. coli* (Table 1). Additionally, many of these hybrids exhibit favorable pharmacokinetic properties, with no obvious toxicity toward human hepatocyte cells (Cui et al., 2014).

Quinolone dimer, including quinolone–fluoroquinolone and fluoroquinolone–fluoroquinolone conjugates, have also been reported (Panda et al., 2015; Ross et al., 2015). However, quinolone–fluoroquinolone conjugates with amino acid linkers have shown poor antimicrobial activity against *P. aeruginosa* (Panda et al., 2015). Meanwhile, fluoroquinolone–fluoroquinolone conjugates with variable linkers—such as polyethylene glycol chains, basic amines, and aryl linkers—exhibited significantly low MIC values against *E. coli* and *P. aeruginosa* (0.06 and 0.125 μg/ml, respectively) (Ross et al., 2015).

In summary, various quinolone-containing antibiotic hybrids have been synthesized, and many have demonstrated significant good antibacterial activity against a range of Gram-negative bacteria (Table 1). Notably, fluoroquinolone-oxazolidinone and fluoroquinolone–rifampicin hybrids have progressed into clinical trials (Koh et al., 2023). However, these compounds are being evaluated primarily for the treatment of bacterial infections caused by Gram-positive bacteria (Koh et al., 2023). To properly assess the therapeutic potential of hybrid antibiotics, studies examining in vivo efficacy and safety using animal models for infection are required. However, these studies were not performed in most hybrid antibiotics in this section. Therefore, on the basis of the present study alone, the efficacy of hybrid antibiotics cannot be clearly determined.

## Other Antibiotic Hybrids

In addition to quinolone-containing antibiotic hybrids, a wide range of hybrid compounds with diverse drug combinations have been described (Table 1). Polymyxins are membrane-targeting decalipopeptide antibiotics that disrupt bacterial membranes and interfere with proton motive forces (Velkov et al., 2013). Polymyxin B_3_-based hybrid compounds fused with tobramycin, an aminoglycoside antibiotic, have been reported (Domalaon et al., 2017). These hybrid compounds show moderate antibacterial activity against various Gram-negative pathogens, including *E. coli*, *K. pneumoniae*, *A. baumannii*, and *P. aeruginosa* (Table 1). Notably, they exhibit potent activity against clinical isolates of carbapenem-resistant and colistin-resistant *P. aeruginosa* and show strong synergy with minocycline, rifampicin, and vancomycin against multidrug-resistant *P. aeruginosa* (Domalaon et al., 2017). Polymyxin E-vancomycin hybrid conjugates have also been reported (van Groesen et al., 2021). These compounds showed very potent antibacterial activity against vancomycin-resistant Gram-positive pathogens but demonstrate only moderate activity against Gram-negative pathogens, including *K. pneumoniae*, *A. baumannii*, and *P. aeruginosa* (Table 1). Similarly, a vancomycin-based hybrid compound fused to nisin, a natural polycyclic antibacterial peptide, shows moderate antibacterial activity against *K. pneumoniae* and *M. catarrhalis* (Arnusch et al., 2008), likely due to the high molecular weight of vancomycin.

A rifamycin-based hybrid fused to nitroimidazole (TNP-2198) showed a significantly low MIC value (0.004 μg/ml) against *H. pylori* (Ma et al., 2022). The nitroimidazole moiety contains a nitro group and an imidazole ring, as found in metronidazole. TNP-2198 has also demonstrated potent activity against *H. pylori* strains resistant to both rifamycin and nitroimidazole (Table 1). This compound is currently undergoing Phase II clinical trials for the treatment of *H. pylori* and *C. difficile* infections as well as bacterial vaginosis (Koh et al., 2023; Ma et al., 2022).

Further, two antibiotic hybrids containing aminoglycoside antibiotics have also been synthesized (Findlay et al., 2012; Hanessian et al., 2011). Neomycin B hybrids are linked to triclosan, an antibacterial and antifungal agent that inhibits bacterial fatty acid synthesis. They show good antibacterial activity against *E. coli*, *K. pneumoniae*, and *A. baumannii*, but not against *P. aeruginosa* (Findlay et al., 2012). In contrast, sisomicin-based hybrid compounds fused with gentamicin, another aminoglycoside antibiotic, demonstrate good antibacterial activity against *P. aeruginosa*, along with *E. coli* and *K. pneumoniae* (Hanessian et al., 2011).

Linezolid-based hybrid compounds are fused to sparsomycin, a nucleoside analog of uracil that binds to the 50S ribosomal subunit and inhibits bacterial protein synthesis via peptidyl transferase inhibition (Ottenheijm et al., 1986). These compounds show moderate antibacterial activity against *H. influenzae* (Zhou et al., 2008). Another oxazolidinone-based hybrid linked to cephalosporin has also been described (Liu et al., 2018). To enhance cellular uptake of hybrid antibiotics, siderophores—high-affinity iron-chelating compounds (Schalk, 2025)—have been incorporated into these hybrid antibiotics. Siderophore-mediated active transport resulted in low MIC values against *E. coli*, *A. baumannii*, and *P. aeruginosa* (0.025, 0.4, and 0.4 μg/ml, respectively) (Liu et al., 2018). In the presence of β-lactamases, such as Acinetobacter-derived cephalosporinase-1 (ADC-1), intracellular release of oxazolidinone upon cephalosporin cleavage allows the freed oxazolidinone to inactivate its target. Consequently, the oxazolidinone–cephalosporin–siderophore conjugate demonstrated a relatively low MIC (6 μg/ml) against *A. baumannii* expressing ADC-1 (Table 1).

More recently, mupirocin-based hybrid compounds fused to holomycin, a dithiolopyrrolone antibiotic (Liras, 2014), have been reported (Johnson et al., 2024). Mupirocin is a pseudomonic acid antibiotic that inhibits isoleucine-tRNA ligase in bacteria, and is generally ineffective against the most common Gram-negative bacteria owing to its poor outer membrane penetration (Khoshnood et al., 2019). However, mupirocin–holomycin conjugates show enhanced antibacterial activity against *E. coli* compared with mupirocin alone (Johnson et al., 2024).

## Mechanisms Underlying Synergistic Effects of Combination Therapy

Although hybrid antibiotics and combination therapy have distinct pharmacokinetic and pharmacodynamic properties (Fig. 1), both have in common that they use two antibacterial moieties. Therefore, insights derived from combination therapy may aid in the development of effective hybrid antibiotics. In this context, we will explore combination therapy. The fractional inhibitory concentration index (FICI) is commonly used to measure the interactions between two antibiotics (Odds, 2003). FICI values of ≤ 0.5, 1, and ≥ 4 indicate synergy, no interaction, and antagonism, respectively (Odds, 2003). Although numerous antibiotic combinations with FICIs of ≤ 0.5 have been reported, the underlying molecular mechanisms responsible for their synergy have not often been elucidated. Here, we summarize recent studies that have revealed the molecular basis of synergistic interactions between antibiotic combinations (Table 2).

Combination therapy with trimethoprim and sulfamethoxazole, also known as cotrimoxazole, is widely used to treat various bacterial and certain fungal infections (Smilack, 1999). Surprisingly, the molecular basis of their synergistic effect has been clarified only recently (Minato et al., 2018). The two antibiotics inhibit sequential steps in tetrahydrofolate biosynthesis: trimethoprim blocks the conversion of dihydrofolate to tetrahydrofolate, whereas sulfamethoxazole inhibits dihydropteroate production from *p*-aminobenzoic acid and 6-hydroxymethyl-7,8-dihydropterin pyrophosphate (Minato et al., 2018). Generally, the potent synergy between the two antibiotics is believed to be caused by the sequential inhibition of adjacent biosynthetic steps, despite limited experimental evidence. A recent report showed that other antibiotic combinations inhibiting sequential steps in tetrahydrofolate biosynthesis do not demonstrate a synergistic effect; only a mixture of trimethoprim and sulfamethoxazole showed this synergy (FICI = 0.31) (Minato et al., 2018). Mechanically, sulfamethoxazole potentiates trimethoprim by limiting de novo dihydrofolate production, while trimethoprim enhances sulfamethoxazole activity by inhibiting dihydropterin pyrophosphate synthesis (Minato et al., 2018). These findings indicate that the synergy between the two antibiotics may be derived from delicate physiological mechanisms.

Nitrofurantoin is a nitrofuran antibiotic commonly used to treat uncomplicated urinary tract infections (Mahdizade Ari et al., 2023). It shows strong synergistic interactions with amikacin against *E. coli* (FICI = 0.375) (Ren et al., 2023). Amikacin is an aminoglycoside antibiotic that induces mistranslation of messenger RNA, leading to the accumulation of misfolded proteins (Mingeot-Leclercq et al., 1999). These mistranslated proteins cause bacterial envelope stress, constitutively activating the CpxAR two‐component system. Activation of Cpx signaling stimulates the expression of NfsAB, a major bacterial nitroreductase, through the SoxS/MarA regulons. Nitroreductases overexpression generates lethal reactive intermediates via nitroreduction and promotes prodrug activation of nitrofurantoin (Ren et al., 2023). These results show that stress-induced changes in gene expression can enhance the antibacterial activity of specific antibiotics.

Antibiotics uptake and efflux across bacterial membranes play a pivotal role in antibacterial efficacy, particularly in Gram-negative bacteria that block penetration of high-molecular weight antibiotics through their outer membrane (O'Shea and Moser, 2008). Several studies have reported that antibiotic synergy is mediated by augmented penetration of antibiotics (Table 2). For example, the nalidixic acid–tetracycline combination shows strong synergy against both multidrug-resistant *E. coli* (FICI = 0.25–0.5) and multidrug-resistant *A. baumannii* (FICI = 0.1875–0.5) (Gaurav et al., 2021). This synergy is mediated by the enhanced uptake and reduced efflux of tetracycline by nalidixic acid (Gaurav et al., 2021). Notably, the nalidixic acid–tetracycline combination did not show synergy against antibiotic-susceptible *A. baumannii* and *E. coli* isolates (Gaurav et al., 2021).

Flavomycin is a widely used antibiotic feed additive in the livestock industry that inhibits bacterial cell wall synthesis (Ostash et al., 2010). Colistin, also known as polymyxin E, is a polycationic cyclic polypeptide antibiotic that binds to lipopolysaccharides and phospholipids in the outer cell membrane of Gram-negative bacteria. This leads to disruption of the outer cell membrane (Li et al., 2006). The flavomycin–colistin combination shows strong synergy against both *E. coli* (FICI = 0.19–0.49) and *mcr-1*-positive *E. coli* (FICI = 0.023–0.375) (Huang et al., 2024). This synergy manifests as augmented penetration of flavomycin by colistin, leading to increased intracellular accumulation of flavomycin and improved bacterial killing (Huang et al., 2024).

Similarly, the novobiocin–colistin combination exhibits strong synergy against colistin-resistant *A. baumannii* (FICI = 0.129) and *K. pneumoniae* (FICI = 0.012) (May et al., 2017). Although this synergy is initially believed to result from enhanced novobiocin penetration by colistin, due to high molecular weight of novobiocin (612 Da), similar to flavomycin (1583 Da), subsequent studies have shown that it instead results from novobiocin-mediated potentiation of colistin activity (Mandler et al., 2018; Mattingly et al., 2020; May et al., 2017). Notably, novobiocin, an aminocoumarin antibiotic that inhibits bacterial DNA gyrase (Lawson and Stevenson, 2012), also binds and activates the ATPase LptB that powers lipopolysaccharide transport, which enhances polymyxin activity (Mandler et al., 2018; May et al., 2017). Novobiocin derivatives were synthesized to separate DNA gyrase inhibition from LptB stimulation. Although one of the derivatives retained only LptB-stimulatory activity, it continued to demonstrate strong synergy with polymyxin by binding to LptB and stimulating lipopolysaccharide transport (Mandler et al., 2018).

More recently, the synergistic mechanism underlying hygromycin A–macrolides combinations was revealed through structural and biochemical experiments (Chen et al., 2023). Hygromycin A, also known as totomycin, is a modified cinnamic acid antibiotic containing a furanose sugar and aminocyclitol moiety. It prevents the formation of new peptide bonds by targeting the catalytic peptidyl transferase center (PTC) of the 50S large ribosomal subunit, and selectively acts against *Borrelia burgdorferi*, the causative agent of Lyme disease (Leimer et al., 2021). Macrolides are a class of natural antibiotics with a large macrocyclic lactone ring attached to deoxy sugars (Zhanel et al., 2001). These antibiotics inhibit bacterial ribosomal translation by targeting the nascent peptide exit tunnel (NPET) of the 50S large ribosomal subunit (Vazquez-Laslop and Mankin, 2018). The hygromycin A–macrolides combination shows moderate synergy (FICI = 0.5) against *B. burgdorferi* (Table 2). Among the various PTC-targeting antibiotics, such as hygromycin A, clindamycin, and linezolid, only hygromycin A can simultaneously bind the 70S ribosome with NPET-targeting macrolides without a steric clash. Hygromycin binding slows the dissociation of macrolides from the ribosome, which potentiates the efficacy of macrolides in bacterial growth inhibition and early killing (Chen et al., 2023). Collectively, these reports demonstrate that antibiotic synergy is mediated by various complex and highly specific mechanisms.

## Conclusion

Owing to the challenges associated with the development of novel antibiotics, alternative treatment strategies using currently available antibiotics have been extensively studied. The development of dual-acting hybrid antibiotics has been a long-standing research direction for improving the antibacterial efficacy of existing antibiotics. Among these hybrid compounds, fluoroquinolone–oxazolidinone, fluoroquinolone–rifampicin, and rifamycin–nitroimidazole hybrids have entered clinical trials (Table 1) (Koh et al., 2023). However, as most antibiotics do not have outer membrane-destabilizing activity exhibited by colistin, an increase of the molecular weight by covalent fusion of two antibiotics can block outer membrane penetration in Gram-negative bacteria. Consequently, most hybrid compounds currently in clinical trials have been tested primarily against Gram-positive bacteria. Only rifamycin–nitroimidazole hybrids are currently in Phase II clinical trials for the treatment of *H. pylori* infections (Koh et al., 2023). Therefore, combining antibiotics that exhibit strong synergy may provide some assistance in overcoming the unavoidable disadvantage of increased molecular weight. Unfortunately, the molecular mechanisms underlying the synergistic effects between currently available antibiotics remain poorly understood. Consequently, further efforts are required to elucidate these mechanisms. Although synergy between two antibiotics does not inherently compensate for impermeability or pharmacokinetic constraints caused by increased molecular weight, the pharmacodynamic effects conferred by synergy can positively affect the efficacy of hybrid antibiotics. Therefore, the elaborate design of dual-acting hybrid antibiotics informed by well-defined synergistic mechanisms can substantially enhance the successful development of hybrid antibiotics for the treatment of infections caused by Gram-negative bacteria. This perspective can be one of useful strategies for achieving the development of effective hybrid antibiotics. As physicochemical factors, including polarity, charge distribution, conformational flexibility, amphiphilicity, and susceptibility to efflux, are also important for uptake of hybrid antibiotics, modification of physicochemical properties or combination therapy with membrane-active antimicrobial adjuvant to enhance the efficacy of hybrid antibiotics is also one of useful strategies for developing effective hybrid antibiotics.

## Figures and Tables

**Table 1.** Antibiotic hybrids against Gram-negative pathogens

Hybrid antibiotics	Target pathogens	MIC (mg/ml)	MIC in the resistant mutant strains	Development stage	Comments	Reference
Ciprofloxacin–cephalosporin	*E. coli*	0.18	NDM-expressing *E. coli*: 0.05	In vitro bacteria assay		Evans et al. (2019)
Ciprofloxacin–neomycin	*E. coli*	0.75–6	Neomycin-*resistant E. coli*: 6	In vitro bacteria assay		Pokrovskaya et al. (2009)
Ciprofloxacin–kanamycin	*E. coli*	0.28–12	Kanamycin-*resistant E. coli*: 0.1–3.0	In vitro bacteria assay	The hybrids had significantly lower resistance frequencies in *E. coli* than each drug alone or their combination mixture.	Shavit et al. (2017)
Ciprofloxacin–tobramycin	*P. aeruginosa*	4	ND	In vitro bacteria assay	The compound showed strong synergy with fluoroquinolone against multidrug-resistant *P. aeruginosa*, *A. baumannii*, and *K. pneumoniae*.	Gorityala et al. (2016)
Ciprofloxacin–tobramycin	*P. aeruginosa*	64	ND	In vitro bacteria assay	The compound showed strong synergy with ciprofloxacin, levofloxacin, and moxifloxacin against multidrug-resistant *P. aeruginosa*.	Dhiman et al. (2023)
Ciprofloxacin–erythromycin	*H. influenzae*	4	ND	In vitro bacteria assay		Hutinec et al. (2010)
Ciprofloxacin–erythromycin	*H. influenzae, M. catarrhalis*	*H. influenzae*: 2	ND	In vitro bacteria assay		Ma et al. (2019)
		*M. catarrhalis*: 0.5				
Quinolone–telithromycin	*H. influenzae, E. coli, K. pneumoniae*	*H. influenzae*: 2	ND	In vitro bacteria assay		Fan et al. (2020)
		*E. coli*: 8				
		*K. pneumoniae*: 16				
Quinolone–telithromycin	*H. influenzae, M. pneumoniae*	*H. influenzae*: 4 *M. pneumoniae*: 0.008	Erythromycin-resistant *M. pneumoniae*: 8	In vitro bacteria assay		Liu et al. (2022)
Ciprofloxacin–oxazolidinone	*H. influenzae, E. coli*	*H. influenzae*: 0.06	ND	In vitro bacteria assay		Hubschwerlen et al. (2003a)
		*E. coli*: 1				
Ciprofloxacin–oxazolidinone	*H. influenzae, E. coli*	*H. influenzae*: 0.03	ND	In vitro bacteria assay		Hubschwerlen et al. (2003b)
		*E. coli*: 0.5				
Fluoroquinolone–oxazolidinone	*H. influenzae, E. coli, P. aeruginosa*	*H. influenzae*: 0.016	ND	In vitro bacteria assay		Liu et al. (2019)
		*E. coli*: 1				
		*P. aeruginosa*: 16				
Fluoroquinolone–oxazolidinone (cadazolid)	*E. coli*	*E. coli*: 8	ND	Discontinued in 2018 after two Phase III trials		Scaiola et al. (2019)
Fluoroquinolone–rifampicin	*H. influenzae, E. coli, N. gonorrhoeae, H. pylori*	*H. influenzae*: 0.012	ND	Phase II for acute bacterial skin and skin structure infection		Yuan et al. (2020)
		*E. coli*: 0.25				
		*N. gonorrhoeae*: 0.06				
		*H. pylori*: 0.12				
Fluoroquinolone–kanglemycin	*E. coli*	4	ND	In vitro bacteria assay		Peek et al. (2022)
Ciprofloxacin–sulfonamide	*E. coli*	0.013	ND	In vitro bacteria assay		Ibrahim et al. (2022)
Quinolone–trimethoprim	*P. aeruginosa, K. pneumoniae*	*P. aeruginosa*: 8–16	ND	In vitro bacteria assay		Karoli et al. (2012)
		*K. pneumoniae*: 8				
Quinolone–metronidazole	*P. aeruginosa, E. coli*	*P. aeruginosa*: 2	ND	In vitro bacteria assay	Several compounds showed appropriate ranges to pharmacokinetic behaviors and no obvious toxicity to human hepatocyte cells.	Cui et al. (2014)
		*E. coli*: 0.5				
Quinolone–metronidazole	*P. aeruginosa, E. coli*	*P. aeruginosa*: 0.25	ND	In vitro bacteria assay		Zhang et al. (2015)
		*E. coli*: 0.25				
Quinolone–fluoroquinolone	*P. aeruginosa*	312.5	ND	In vitro bacteria assay		Panda et al. (2015)
Ciprofloxacin dimer	*E. coli, P. aeruginosa*	*P. aeruginosa*: 0.125	ND	In vitro bacteria assay		Ross et al. (2015)
		*E. coli*: 0.03				
Polymyxin B_3_–tobramycin-	*E. coli, K. pneumoniae, A. baumannii, P. aeruginosa*	*P. aeruginosa*: 2	Carbapenem-resistant MDR/XDR *P. aeruginosa*: 2–16	In vitro bacteria assay	The compound showed strong synergy with minocycline, rifampicin, and vancomycin against multidrug-resistant *P. aeruginosa*.	Domalaon et al. (2017)
		*A. baumannii*: 16	Colistin-resistant *P. aeruginosa*: 4–32			
		*E.* coli: 8				
		K*. pneumoniae*: 128				
Polymyxin E–vancomycin (vancomyxin)	*K. pneumoniae, A. baumannii, P. aeruginosa*	*K. pneumoniae*: 8	ND	In vitro bacteria assay		van Groesen et al. (2021)
		*P. aeruginosa*: 16				
		*A. baumannii*: 16				
Vancomycin–nisin	*K. pneumoniae, M. catarrhalis*	*K. pneumoniae*: 16	ND	In vitro bacteria assay		Arnusch et al. (2008)
		*M. catarrhalis*: 16				
Rifamycin–nitroimidazole (TNP-2198)	*H. pylori*	*H. pylori*: 0.004	Rifamycin-resistant *H. pylori*: 0.5	Phase II for *H. pylori*, vaginosis and CDAD ongoing since 2021/2022		Ma et al. (2022)
			Rifamycin- and metronidazole-resistant *H. pylori*: 0.5			
Neomycin B–triclosan	*E. coli, K. pneumoniae, A. baumannii, P. aeruginosa*	*E. coli*: 0.25	ND	In vitro bacteria assay		Findlay et al. (2012)
		*K. pneumoniae*: 1				
		*P. aeruginosa*: 64				
		*A. baumannii*: 8				
Sisomicin–gentamicin	*E. coli, K. pneumoniae, P. aeruginosa*	*E. coli*: 0.5	ND	In vitro bacteria assay		Hanessian et al. (2011)
		*K. pneumoniae*: 0.25				
		*P. aeruginosa*: 0.5				
Linezolid–sparsomycin	*H. influenzae*	4	ND	In vitro bacteria assay		Zhou et al. (2008)
Oxazolidinone–cephalosporin–siderophore	*E. coli, A. baumannii, P. aeruginosa*	*E. coli*: 0.025	ADC-1-expressing *A. baumannii*: 6	In vitro bacteria assay		Liu et al. (2018)
		*A. baumannii*: 0.4				
		*P. aeruginosa*: 0.4				
Mupirocin–holomycin	*E. coli*	64	ND	In vitro bacteria assay		Johnson et al. (2024)

b Not determined.

**Table 2.** Combination therapies against Gram-negative pathogens

Combination antibiotics	Target pathogens	FICI	Synergy mechanisms	Clinical uses	Comments	Reference
Trimethoprim and sulfamethoxazole	*E. coli*	0.31	Mutual potentiation (Sulfamethoxazole potentiates trimethoprim by limiting de novo dihydrofolate production and trimethoprim potentiates sulfamethoxazole activity through inhibition of dihydropterin pyrophosphate synthesis)	Clinical use		Minato et al. (2018)
Amikacin and nitrofurantoin	*E. coli, K. pneumoniae*	*E. coli*: 0.375	Amikacin induces bacterial envelope stress by introducing mistranslated proteins, thereby constitutively activating the CpxAR two‐component system. The activation of Cpx signaling stimulates the expression of bacterial major nitroreductases (NfsAB). Nitroreductases overexpression generates considerable quantity of lethal reactive intermediates via nitroreduction and promotes the prodrug activation of nitrofurantoin.	No		Ren et al. (2023)
		*K. pneumoniae*: 0.5				
Nalidixic acid and tetracycline	Multidrug-resistant *A. baumannii* and *E. coli*	Multidrug-resistant *A. baumannii*: 0.1875–0.5	Enhanced uptake and reduced efflux of tetracycline by nalidixic acid explain the basis of synergy between nalidixic acid and tetracycline.	No	Nalidixic acid and tetracycline combination did not display synergy against susceptible *A. baumannii* and *E. coli* isolates.	Gaurav et al. (2021)
Flavomycin and colistin	*E. coli*, *mcr-1*-positive *E. coli*	*E. coli*: 0.19–0.49	The synergy is manifested as an augmented penetration of the *E. coli* OM by colistin, leading to increased intracellular accumulation of flavomycin and enhanced cell killing thereafter.	No		Huang et al. (2024)
Novobiocin and colistin	Colistin-resistant *A. baumannii* and *K. pneumoniae*	Colistin-resistant *A. baumannii*: 0.129	Novobiocin binds and activates the ATPase LptB that powers lipopolysaccharide transport, which enhances polymyxin activity.	No		May et al. (2017); Mandler et al. (2018); Mattingly et al. (2020)
		Colistin-resistant *K. pneumoniae*: 0.012				
Hygromycin A and macrolides	*B. burgdorferi*	0.5	Hygromycin A cooperatively binds ribosomes with nascent peptide exit tunnel-targeting macrolides and slows down their dissociation, which potentiates macrolide’s efficacy in bacterial growth inhibition and early killing.	No		Chen et al. (2023)

b Not determined.
